# Elevated circulating group-2 innate lymphoid cells expressing activation markers and correlated tryptase AB1 levels in active ascariasis

**DOI:** 10.3389/fimmu.2024.1459961

**Published:** 2024-10-25

**Authors:** Juan-Felipe López, Josefina Zakzuk, Pattraporn Satitsuksanoa, Ana Lozano, Laura Buergi, Anja Heider, Juan Carlos Alvarado-Gonzalez, Huseyn Babayev, Cezmi Akdis, Willem van de Veen, Luis Caraballo, Mübeccel Akdis

**Affiliations:** ^1^ Swiss Institute of Allergy and Asthma Research (SIAF), University of Zurich, Davos, Switzerland; ^2^ Institute for Immunological Research, University of Cartagena, Cartagena, Colombia

**Keywords:** ascariasis, helminth, IgE, immune regulation, innate lymphoid cells, type-2 immunity, Tryptase AB1

## Abstract

**Introduction:**

*Ascaris lumbricoides* infection is one of the most common soil-transmitted helminthiasis and IgE response to this helminth may increase the risk of asthma, bronchial hyperreactivity and atopy. There is not enough evidence showing the role of group-2 innate lymphoid cells (ILC2) in the pathogenesis of helminth infections in humans. Here, we aimed to investigate and characterize the influence of *Ascaris lumbricoides* infection on circulating ILCs in endemically exposed subjects.

**Methods:**

Non-infected (NI; n=16) and Ascaris-infected (AI; n=16) subjects from an endemic area were included. Two consecutive stool samples from each subject were examined by Kato-Katz to define parasite infection. Antibodies to the ABA-1 antigen of Ascaris and Ascaris extract were measured by ELISA. ILC subsets and their activation markers (CD25, CD69, thymic stromal lymphopoietin receptor (TSLPR) were evaluated in its PBMC by flow cytometry. Proximity extension assay (PEA) was performed to explore plasma proteins associated to infection.

**Results:**

No significant differences in the relative or absolute frequencies of total ILCs, ILC1, ILC2 and ILC3 cells were observed regarding the infection status. However, within AI group, IgE-sensitized subjects to ABA-1 had higher frequencies and counts of ILC2 (p<0.05). Frequencies of CD25+, CD69+ and TSLPR+ ILC2 were higher in AI compared to the NI (p<0.01). Additionally, egg burden was positively correlated with CD69+ ILC2 frequencies (r=0.67; p=0.005). Tryptase alpha/beta 1 (TPSAB1), GP6 and several plasma proteins associated with cell growth and granulocyte chemotaxis were highly expressed in the AI group (p<0.05). Interestingly, TPSAB1 levels were positively correlated with ILC2 expressing activation markers frequencies, egg burden and IgE levels against Ascaris.

**Discussion:**

Ascaris infection is associated with increased expression of ILC2 activation markers and TPSAB1, which may contribute to the type-2 response.

## 1 Introduction

*Ascaris lumbricoides* infection is one of the most prevalent and neglected tropical helminthiasis worldwide (global prevalence around 11.9%) (1). In addition, several studies have shown that sensitization to its components is associated with indicators of poorly hygienic conditions and asthma risk (2–8). Similar to allergy, helminth infection induces a strong type-2 (T2) response that is orchestrated by alarmins, cytokines, and cells from innate and adaptive immune systems (9).

Innate lymphoid cells (ILCs) are mainly tissue-resident lymphocytes that do not express antigen-recognition receptors (10). They produce cytokines essential for the maintenance of tissue homeostasis and communication between the innate and adaptive immune systems (10). Recent studies have demonstrated the importance of ILCs and alarmins (IL-25, IL-33) in the promotion of T2 immunity and inflammation following helminth infection or allergen exposure (11–14). ILC2s, a subtype of ILCs discovered in a mouse model of helminth infection, are essential for Th2-differentiation and worm expulsion due to their role in the production of IL-4 and IL-13, respectively (15–18). Observations from human studies in *Schistosoma haematobium* and filarial infection have demonstrated that ILC frequencies fluctuate according to the infection status (19, 20). However, these findings are heterogeneous and were reported in different helminth species.

Since mice are not a natural host for Ascaris spp. and larvae do not develop into adult stages, there is no clear evidence that *A. lumbricoides* induces changes in ILCs in this species. Due to the strong promoting effect of Ascaris on T2 responses, it is hypothesized that this infection may induce ILC2 development and activation. Epithelial alarmins stimulation and different status of disease can induce changes in the different surface markers associated with this population. Several studies in different models have shown that activation of ILCs could be represented by global lymphoid cell activation markers like CD69 (21–23) and CD25 (24, 25), and thymic stromal lymphopoietin receptor (TSLPR) represents a proxy of T2 response susceptibility of this cell subset (26, 27).

Understanding how helminths shape the immune system provides the opportunity to gain insight into natural T2 responses and how they might be regulated. Additionally, exploration of helminth targets in the immune system may help to elucidate the mechanisms by which potential vaccine candidates fail to provide sustained immunity (28). Hence, this study aimed to investigate and characterize specifically the influence of helminth infection by *A. lumbricoides* on circulating ILCs of exposed subjects living in an endemic town of Colombia.

## 2 Materials and methods

### 2.1 Study subjects

From March 2022 to September 2022, subjects were recruited through a cross-sectional survey in Santa Catalina, a small tropical farming/fishing town in the North of Colombia (10° 36′ 0″ N, 75° 18′ 0″ W) that includes five villages (153 km2, 12.500 inhabitants, no sewage) where *Ascaris lumbricoides* infection is endemic (prevalence of 63%) (7, 29). In a last survey, half of its inhabitants had at least one unsatisfied basic need, only 4.5% of the population has a sewage system and 56% has tap water.

Eligible participants were adults (between 18 to 75 years old), who had lived in the town for at least 2 years. They were visited by a trained physician, who examined the subjects, filled out the questionnaires to assess the demographic risk factors for infection, and collected blood and stool samples. Exclusion criteria were co-infection by another helminth, pregnancy, received anti-helminthic in the last 3 months and/or any antecedent of cancer or autoimmune diseases. Controls were non-infected but exposed subjects (inhabitants from this village) with similar sociodemographic conditions, age and sex, without records of worm expulsion in the last 2 years and two consecutives (least than one week apart) negative stool examinations. Once an infected case was identified, a neighbor of the same age and sex without consanguinity (to avoid genetic susceptibility bias) was invited to participate as a control.

Infection status was determined by stool parasitological analyses in two stool samples obtained by spontaneous evacuation on different days and donors were categorized as non-infected (NI) or Ascaris infected (AI). Briefly, all donors were tested under direct fecal smear and Kato-Katz method which allows to determine counting helminth egg per gram (e.p.g) of feces which has a sensitivity and specificity over 90% to detect different soil-transmitted helminth infections (30, 31). If protozoan or helminth parasites different than Ascaris were found, the subject was excluded from the study. Peripheral mononuclear cells (PBMC) were isolated from heparinized blood, cryopreserved and stored in liquid nitrogen until flow cytometry analysis.

The study was approved by the University of Cartagena Ethics Committee (Minutes #12811-2019). Informed consent was obtained from all participants.

### 2.2 Recombinant ABA-1, Ascaris-specific and total IgE antibody detection

*Ascaris lumbricoides* extract was prepared from adult worms after physical homogenization in PBS as reported previously (32). Recombinant ABA-1 was produced in *Escherichia coli* BL21 (Invitrogen Corporation, Carlsbad, CA, USA) as a GST fusion protein. After cleavage with thrombin, a 99% purity product is observed in SDS-PAGE (33). Recombinant ABA-1 and *A. lumbricoides* body extract were coated at 1 µg and 5 µg per well, respectively, on Nunc Maxisorb microtiter plate (Thermo Fisher Scientific, Waltham, MA, USA) and incubated at room temperature (RT) overnight and then blocked with blocking buffer (PBS pH 7.4, 2% BSA, 0.05% Tween 20). Plasma samples diluted 1:10 in blocking buffer were added and incubated for 2 hours at room temperature. Specific IgE was detected using a goat anti-human IgE antibody conjugated with HRP (Bethyl Laboratories, Cat N° A80-108P, Texas, USA). For the detection of specific IgG, goat anti-human IgG-peroxidase (Jackson Immuno Research Europe Ltd, Cambridgeshire, UK) was used. For total IgE detection, high-binding 96-well ELISA plates (Thermo Fisher Scientific, USA) were coated with mouse anti–human IgE clone 14-41 (gift from Christoph Heusser, Novartis, Basel, Switzerland). The plates were blocked and then incubated for 2 hours with 1:100 diluted plasma samples and the 8-step standard curve (300 to 0.13 ng/mL) of human IgE myeloma wild-type (Cat N° 401152; Sigma-Aldrich). Biotinylated mouse anti–IgE clone 6-7 (clone MZ01; Christoph Heusser, Novartis) was used as detection antibody and Extravidin peroxidase (Cat N° E2886; Sigma-Aldrich) was used as an enzymatic reagent (34). ELISAs were developed using tetramethylbenzidine (TMB) substrate (Thermo Fisher Scientific, Waltham, MA, USA) and the reaction was stopped with 1M H2SO4 sulfuric acid. Plates were read at 450 nm by a Mithras LB 940 spectrophotometer (Berthold Technologies, Bad Wildbad, Germany) (35).

### 2.3 Peripheral blood mononuclear cells isolation and cryopreservation

Blood samples were collected in heparinized tubes; subsequently, they were diluted in PBS and centrifuged with Ficoll Histopaque (Sigma Aldrich, St. Louis, MO, USA) to visualize and extract the mononuclear cell layer. Cells were washed three times with PBS/EDTA and resuspended in 1 mL of RPMI 1640 (Sigma Aldrich, St. Louis, MO, USA) supplemented medium and 1 mL of freezing medium (80% inactivated FBS and 20% DMSO). The samples were deposited in cryovials in a freezing container (Thermo Scientific™ Mr. Frosty™ Freezing Container) at -80°C and stored at −196°C in LN2 until analysis.

### 2.4 Flow cytometry

**Figure 1:**
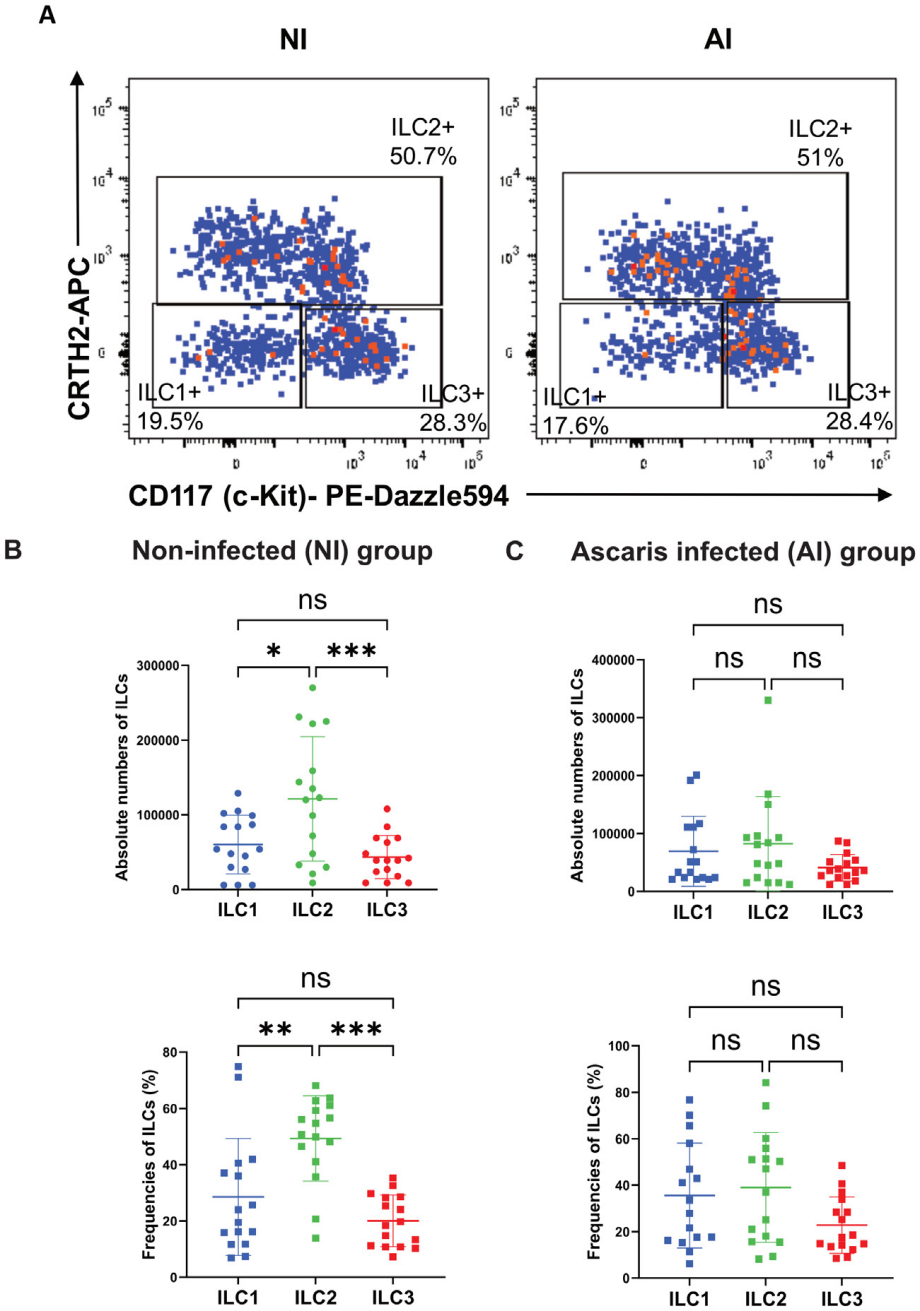
ILCs distribution between NI and AI groups. **(A)** Representative plot of ILC subsets according to infection status. Distribution of frequencies and absolute numbers of ILC subsets (ILC1, ILC2, ILC3) in **(B)** non-infected and **(C)** Ascaris infected. Comparisons were made using Friedman and Dunn’s multiple comparison test. * p<0.05. **p<0.01, *** p<0.001. ns, non-significant.

PBMCs were thawed and stained with a modified panel based from a well-established method for ILCs detection (36, 37). A monoclonal antibody mix containing FITC-labelled surface antibodies was used to exclude dendritic cells (CD11c), plasmacytoid dendritic cells (CD303, CD123), monocytes (CD14), macrophages (CD14), stem cells (CD34), NK cells (CD94), basophils (FceR1a), T lymphocytes (CD3) and B lymphocytes (CD19). CD45, CD161 and CD127 (IL-7Rα) were included to identify the circulating total ILC population (**Supplementary Figure 1A**), and CRTH2 (PGD2 receptor) and CD117(c-kit) to differentiate ILC1s, ILC2s and ILC3s (**Figure 1A**). The full stain panel (**Supplementary Table 1**) also includes TSLPR, and CD69 (c-type lectin protein) and CD25 (IL2Rα) for further phenotyping of ILC2 activation. Isotype and fluorescence minus one (FMO) tubes were used as controls to define the gating strategy (**Supplementary Figure 1B**). Flow cytometric measurements were made according to the compensation matrix of the ILC panel prepared on the BD LSR FORTESA (16-colour analyzer) and were analyzed using the Flowjo LLC Software (Version 10, San Jose, CA, USA).

### 2.5 Proximity extension assay

We used Target 96 Inflammation, Immune Response, Cardiovascular III and Organ Damage panels from Olink Biosciences (Uppsala, Sweden) to characterize 368 proteins in the obtained plasma samples. The PEA method involves binding of antibody-pairs labeled with unique DNA oligonucleotides to the target protein in the samples. The subsequent proximity extension creates unique DNA barcodes which are amplified by real time PCR. Measurements are presented as Normalized Protein eXpression (NPX) values, reflecting relative protein concentration with no absolute quantification, derived from Ct values after data pre-processing to minimize variation (38). Assay characteristics including quality control, detection limits, and measurements of assay performance and validation were done at the Olink Service Provider Lab in Davos, Switzerland. After excluding variables due to >40% missing data (values below the limit of detection; LOD), 239 products were selected for final analysis.

### 2.6 Statistical analysis

Statistical analyses were performed using SPSS version 20 software, GraphPad Prism 9 and R Studio programs. Distribution of variables was examined using visual (histogram and probability graphs) and analytical methods (Kolmogrov-Smirnov/Shapiro-Wilk tests). Descriptive statistics were calculated by using the mean and standard deviation for normally distributed variables and the median and range for non-normally distributed variables.

Groups were compared with each other in terms of total ILCs and ILCs subsets and activated cells by marker expression. Parametric and non-parametric tests were used according to the Gaussian distribution of the variables. The bivariate tests included Pearson’s Chi-square to compare categorical variables and the Mann-Whitney or Kruskal Wallis test to compare continuous variables between two or more groups, respectively.

Parametric statistics was applied for most analysis with data derived from Olink since measurements are expressed as NPX values, which are derived from a log2 transformation of the protein concentration values. This transformation helps to handle a wide range of protein concentrations and can normalize for variations in the assay. T-tests were used for two-group comparisons of proteins (NPX values) between AI and NI subjects. Heatmap was generated using Z-score data as input (which normalized the protein expression across all samples), clustering applied to columns (proteins), and custom colors assigned to the ‘Infected’ and ‘Non-Infected’ status. Euclidean distance metric was used for clustering the proteins. Protein enrichment pathway analysis was done with Enrichr web-server. Nine upregulated proteins were input. Gene Ontology Biological Process 2023 pathways were selected. The top 10 significant pathways were selected according to the highest adjusted p-value and the representation corresponds to -log10 of these values (39).

For correlational analysis of relative counts of ILC2-related variables with Olink proteins, relative cell counts were first log-transformed, and then, Pearson correlation coefficients were obtained. P and adjusted p-values (False Discovery Rate, FDR) are presented. In addition, due to their highly skewed distribution, correlations of egg and specific-antibody data with other variables were determined by Spearman method.

Finally, we explored the importance of different immune variables (selected from previous mentioned analysis) on determining active infection using a random forest model. Corrplot, ggplot2 and pheatmap, random forest, Enhanced volcano packages were used for plotting with R software (version 4.3.0).

## 3 Results

The mean age of the subjects was 52 years proportionally distributed in terms of biological sex in both groups. The median egg burden was 359 eggs per gram (e.p.g). No cases of anemia were detected in the study population. There was not significant difference regarding concentration of total IgE, the strength of the antibody response IgG or IgE to the body-extract of Ascaris or the ABA-1 antigen. The frequency of IgG response to Ascaris body extract was lower in the AI group compared to NI (68.8% vs. 75%). In contrast, IgE response was significantly higher in AI group compared to NI (87% vs. 56%; p<0.05). Likewise, the frequencies of anti-ABA1 IgG (37.5% vs 31.3%) and anti-ABA1 IgE (31% vs 44%) followed the same trend but there were no significant differences between the groups (**Table 1**).

**Table 1:** Clinical features of the study population.

Variable	Non-Infected (n=16)	Ascaris infected (n=16)	p-value
Age (mean ± SD)	52.06 ± 16.5	52.1 ± 16.16	0.99
Female (%)	60%	50%	0.57
Egg count per gram [median (range)]	N/A	359 (1230)	N/A
Total IgE (ng/mL) [median (range)]	52.09 (12.14 – 1205.17)	122.19 (20.82 – 1187.21)	0.16
Anti-Ascaris sIgG (%)	75	68.8	0.69
OD Anti-Ascaris sIgG [median (range)]	0.85 (0.25 – 0.94)	0.76 (0.45 – 0.97)	0.49
Anti-ABA-1 sIgG (%)	37.5	31.3	0.71
OD Anti-ABA-1 sIgG [median (range)]	0.58 (0.37 – 1.14)	0.61 (0.18 – 0.87)	0.93
Anti-Ascaris sIgE (%)	56	87	0.049*
OD Anti-Ascaris sIgE (median, range)	0.13 (0.06 – 0.33)	0.16 (0.08 – 0.23)	0.25
Anti-ABA-1 sIgE (%)	31	44	0.47
OD Anti-ABA-1 sIgE (median, range)	0.10 (0.04 – 0.25)	0.11 (0.04 – 0.30)	0.54

IQR, Interquartil range; OD, Optical density; N/A, Non apply; SD, Standard deviation; sIgE, specific IgE, sIgG, specific IgG.*, Significantly different. Mann-Whithney test was used for 2-groups comparison of continuous variables.

### 3.1 Higher numbers and frequencies of circulating ILC2s are associated with ABA-1 IgE antibody response

**Figure 2:**
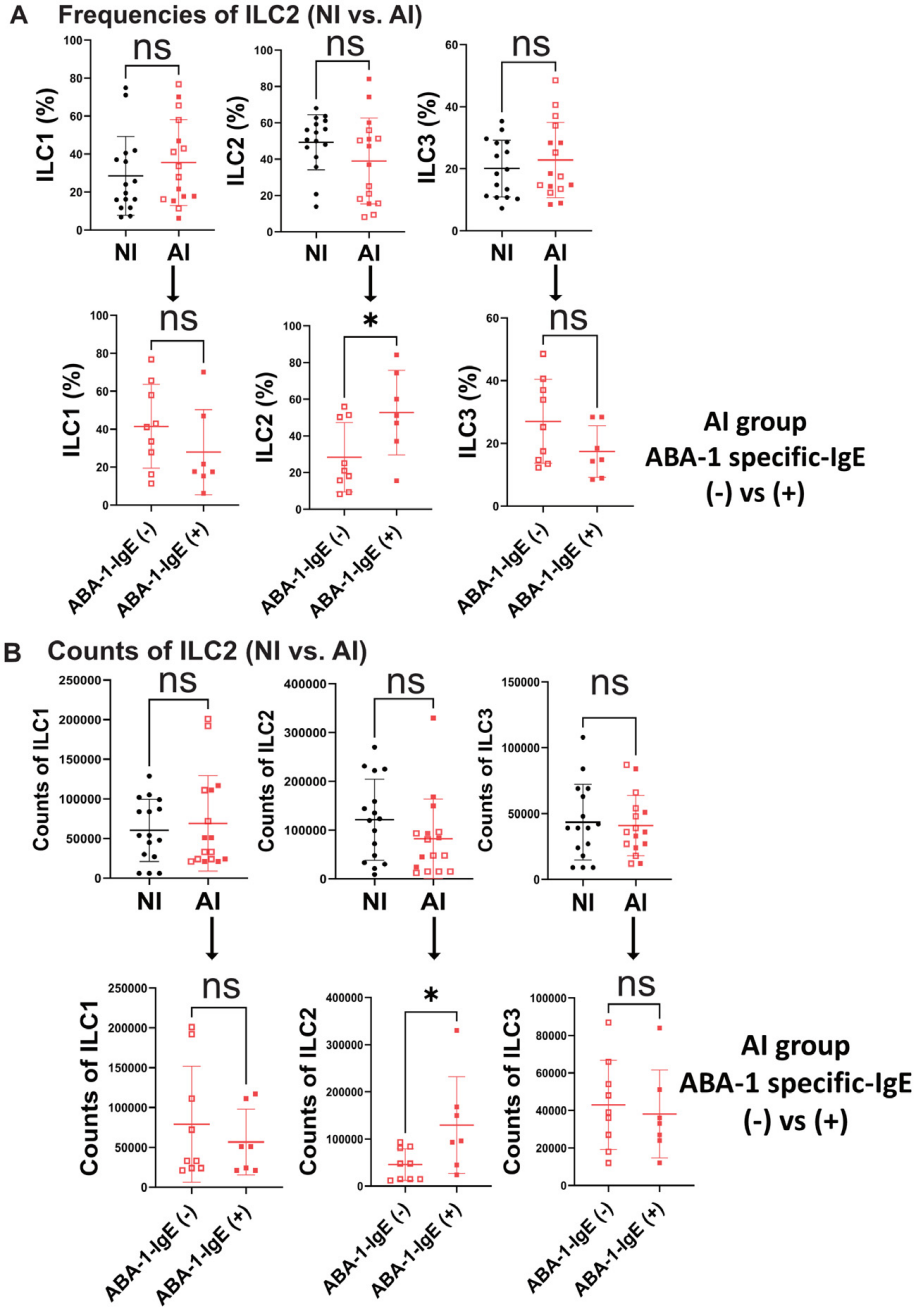
Higher ILC2 Higher frequencies and numbers are associated with anti-ABA-1 IgE sensitization status. Frequencies **(A)** and absolute numbers **(B)** of three mainly ILC subsets (ILC1, ILC2, ILC3) according to Ascaris infection status. Categorization by ABA-1 IgE sensitization is associated with higher ILC2 in the AI group. Comparisons were made using Mann–Whitney according to infection and unpaired T-test according sensitization. * p<0.05. ns, non-significant.

Circulating total ILCs were defined as Lin^−^CD45^+^CD127^+^CD161^+^ (**Supplementary Figure 1A**); whereas c-kit and CRTH2 markers, were used to define ILC subpopulations (ILC1: c-kit^−^CRTH2^−^, ILC2: c-kit^−^CRTH2^+^, and ILC3: c-kit^+^CRTH2^−^) (**Figure 1A**). Although in NI subjects, ILC2s were the most frequent (49.3%) among the three ILC subpopulations (ILC1: 28.5%; ILC3: 20%) (**Figure 1B**), we did not observe significant differences in the relative frequencies or numbers of ILCs among AI individuals (ILC1: 35.6%; ILC2: 39%; ILC3: 22.8%; **Figure 1C**). No significant differences were found in relative or absolute numbers of total ILCs, ILC1, ILC2 or ILC3 between NI and AI subjects. However, we observed a bimodal distribution of ILC2 in AI individuals. Examining different variables, this behavior is likely explained by variations in the IgE response to ABA-1 among infected individuals for the relative and absolute numbers of ILC2. We observed that positive responders in this group had significantly higher frequencies of ILC2 (p < 0.05) (**Figures 2A, B**).

### 3.2 Frequencies of ILC2 expressing activation markers cells are higher in active Ascaris infection and correlated with the egg burden

**Figure 3:**
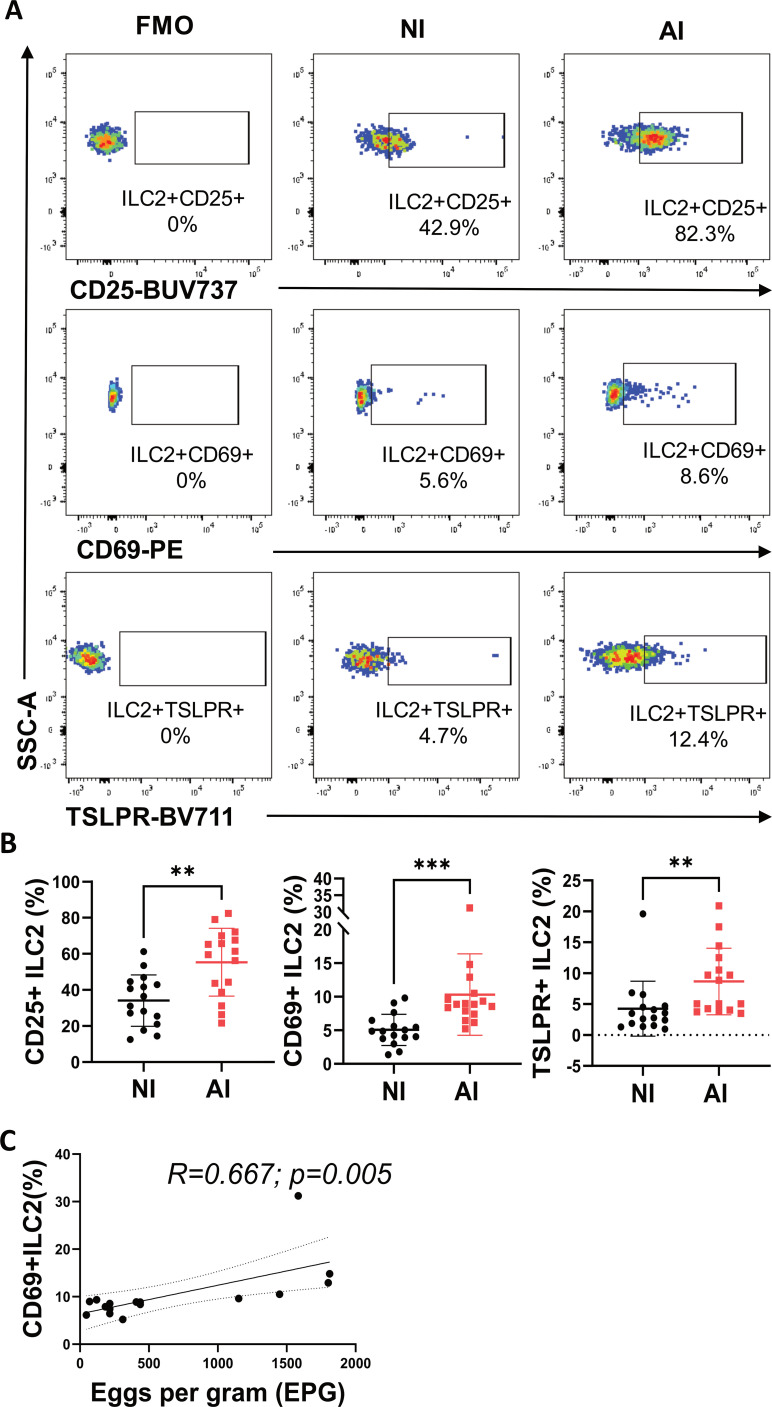
ILC2 expressing activation markers frequencies are associated with Ascaris infection and correlate with the egg burden. **(A)** Representative dot plot of the frequency of activation markers in ILC2s compared between non-infected (NI) and infected (AI) subjects. **(B)** Frequencies of different activation markers in ILC2s according to Ascaris infection status **(C)** Correlation plot between CD69+ILC2 and egg burden. Comparisons were made using the Mann–Whitney test and correlation analysis corresponds to Spearman correlation coefficient (r). ** p<0.01, *** p<0.001.

Surface markers associated with activation and type 2 response (CD25+, CD69+ and TSLPR+) in ILC2 frequencies were also analyzed. In **Figure 3A**, a representative plot of gated CD25+, CD69+ and TSLPR+ ILC2 cells for FMO control as well as infected and NI groups are shown. The frequencies CD25+, CD69+ and TSLPR+ ILC2s were significantly higher in AI compared to the NI controls (**Figure 3B**). Additionally, Ascaris’s egg burden in feces had a positive correlation (r=0.67; p=0.005) with the number of CD69+ ILC2 (**Figure 3C**).

### 3.3 Exploratory analysis of plasma proteins correlated with circulating ILC2s

A correlational analysis of blood ILC2s with circulating proteins in plasma samples, measured through the PEA method, was performed. Before multiple testing correction, there were 50 products associated with either log-transformed % total, CD25+, CD69+ or TSLPR+ ILC2 cells (**Supplementary Figure 2**). Negatively correlations of %ILC2 with granulocytes products and type 2 response proteins like myeloperoxidase (MPO), vascular endothelial growth factor A (VEGFA), amphiregulin (AREG), and chitinase-3 like-protein-1(CHI3L1) were found. Also, %CD25+ ILC2 were negatively correlated with chemokines like CCL3, CCL4, CCL19 and inflammatory cytokines like TNF and IFNγ. On the other hand, TPSAB1 and GP6 were positively correlated with CD25+ and CD69+ ILC2. However, after multiple testing correction (FDR < 0.1), only MMP1 (r=-0.62; p=0.003), VEGFA (r=-0.71; 0.0003) and MPO (r=-0.64; p=0.002) were significantly correlated with %ILC2.

### 3.4 Protein expression profile in active Ascaris infection and involved pathways

**Figure 4:**
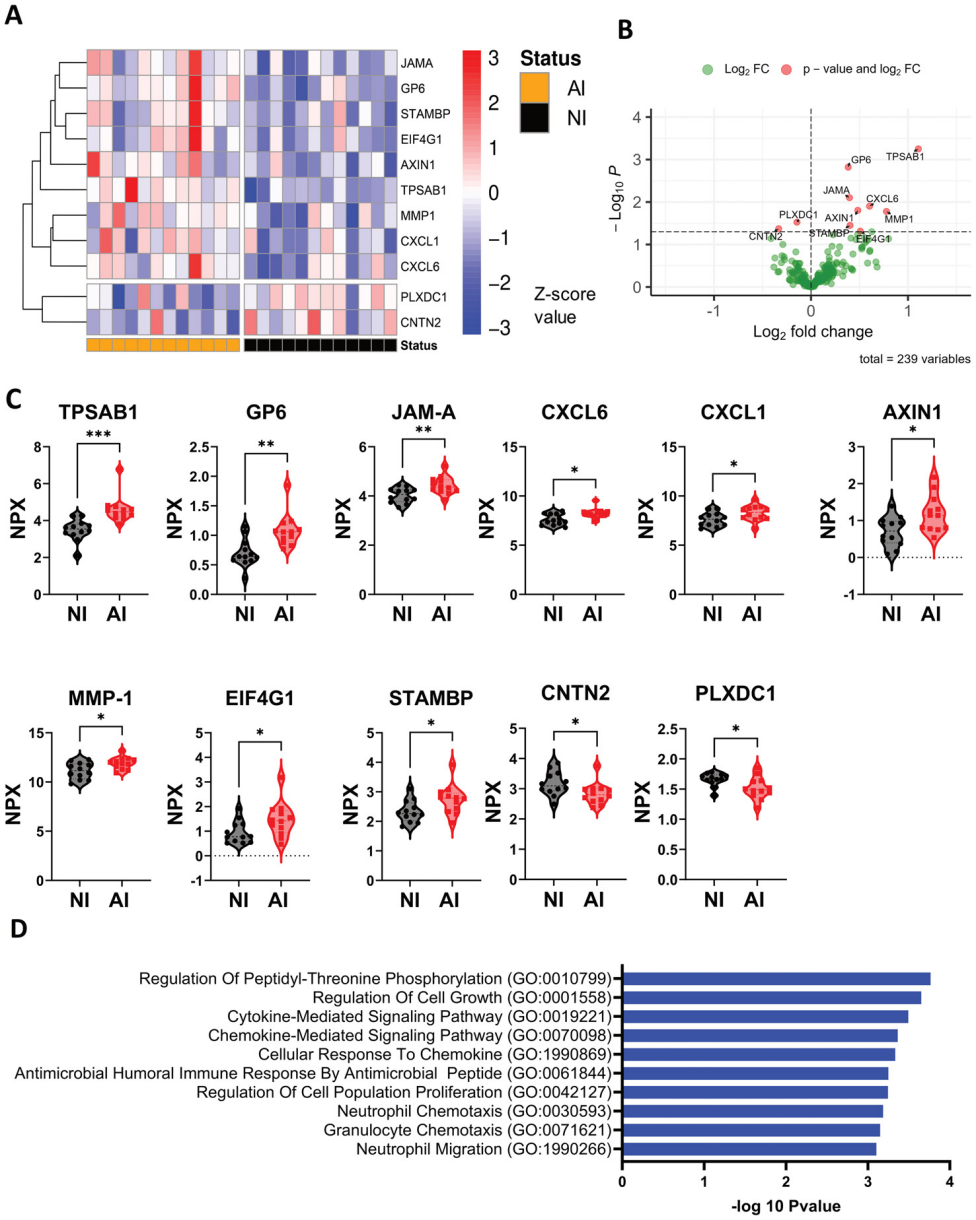
Protein expression profile associated with Ascaris infection and pathways involved during the infection. **(A, B)** The heatmap and volcano plot depicts significant differential protein expression profiles in the NI and AI groups. **(C)** Differential expressed plasma proteins are represented in violin plots: TPSAB1, GP-6, JAM-A, CXCL1, CXCL6, AXIN1, MMP-1, EIF4G1, STAMBP,CNTN2, and PLXDC1; **(D)** Ratio of 10 significant biological processes associated with high expressed protein profile. Comparisons were made using unpaired T-test * p<0.05, ** p<0.01, *** p<0.001.

Eleven proteins that are differentially expressed in Ascaris infection were identified by targeted proteomics performed on plasma samples (**Figures 4A, B**). Tryptase alpha/beta 1 (TPSAB1) from mast cells, glycoprotein 6 (GP-6) which induces activation and aggregation of platelets, and junctional adhesion molecule A (JAM-A), involved in epithelium stability, were significantly high in infected individuals. In addition, the neutrophil (CXCL1) and granulocytes’ (CXCL6) chemotactic proteins, AXIN1 a negative regulator of the wingless-type MMTV integration site family member 1 (WNT) signaling pathway, matrixin 1 (MMP-1) associated to tissue remodeling, eukaryotic translation initiation factor 4 gamma 1 (EIF4G1) and STAMBP, Signal Transducing Adaptor Molecule binding protein were significantly higher expressed in plasma samples of AI compared to NI subjects (**Figure 4C**). In contrast, contactin-2 (CNTN2) a cell adhesion molecule, and plexin domain containing 1(PLXDC1) involved in angiogenesis and endothelial morphogenesis were significantly lower expressed in AI individuals (**Figure 4C**). Highly expressed proteins were included in the pathway analysis, which showed that most of them are involved in granulocyte chemotaxis and migration, regulation of peptidyl-threonine phosphorylation and cell growth, and disassembly of the extracellular matrix and cellular components (**Figure 4D**). In most patients, the alarmins, TSLP and IL-33, as well as Th2 cytokines (IL-4, IL-5 and IL-13) could not be detected in plasma samples. Other proteins related to inflammation, type 1 and type 2 responses and immune regulation pathways did not show any significant differences in regard to infection status (**Supplementary Figure 3**).

### 3.5 Tryptase AB1 levels are associated to ILC2 expressing activation markers, egg burden and IgE response to Ascaris

**Figure 5:**
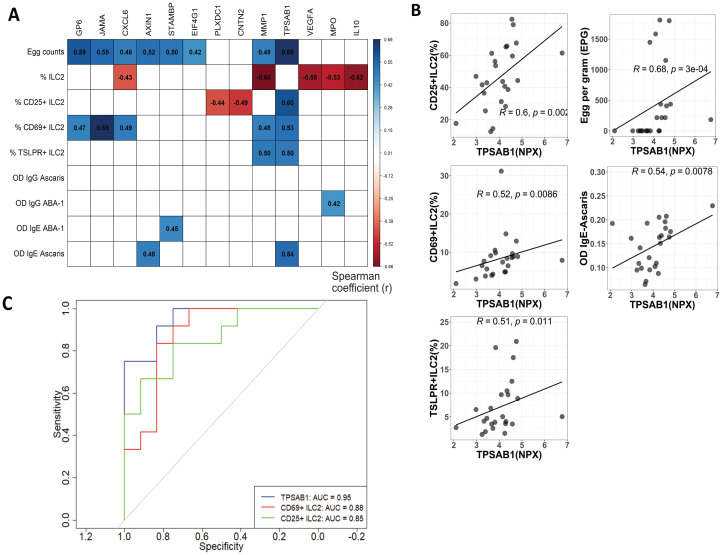
TPSAB1 is correlated to ILC2s expressing activation markers frequencies, egg burden and Ascaris IgE sensitization. **(A)** Correlation values are depicted between NPX of protein expression, humoral parameters, ILC2 and ILC2s expressing activation markers frequencies. Only significant correlations are shown as colored squares. Spearman coefficient (r) values from −0.66 to 0.69 on a red to-blue scale, respectively. **(B)** Plots depicting the positive correlation between TPSAB1 NPX values with frequencies of ILC2s expressing activation markers (CD69+, CD25+ and TSLPR+), egg counts and Ascaris IgE levels **(C)** Receiver operating characteristic (ROC) curves and area under the curve (AUC) obtained from logistic regression models to predict the Ascaris infection. Blue, red and green line corresponding to TPSAB1, CD69+ILC2 and CD25+ILC2, respectively.

Plasma proteins significantly enhanced in active infection were correlated with antibody response intensity, egg burden and frequency of ILC2s and subsets of activation. Remarkably, we found that TPSAB1 levels were positively correlated with frequencies of ILC2 expressing activation markers ILC2 (CD25+, CD69+, TSLPR+), egg counts and intensity of IgE antibody response to Ascaris (r>0.5; p<0.05) (**Figures 5A, B**). IL-10 levels showed a negative correlation with ILC2 frequencies (rho=-0.62; p<0.05) (**Supplementary Figure 4A**).

As an exploratory approach, we also used a random forest model to determine the importance of these variables on predicting *A. lumbricoides* infection. Notably, TPSAB1 values and different phenotypes of ILC2 expressing activation markers had the best performance to predict infection (**Supplementary Figure 4B**). ROC curve analysis found strong predictive performance for TPSAB1 (AUC: 0.95), CD25+ ILC2(AUC: 0.85), and CD69+ ILC2 (AUC: 0.88) over *A. lumbricoides* infection (**Figure 5C**).

## 4 Discussion

As a bridge between the innate and adaptive immune T2 response, ILC2s represent an essential cell subset in the immune response against helminths (40). Several studies, in animal models and humans, have evaluated the role of these cells in the pathophysiology of helminthiasis, however, findings differ among the different infecting helminth species. As a novelty, this study explores the relationship of ILC2 numbers with *A. lumbricoides* infection, finding that ILC2 expressing activation markers are more frequent in infected subjects. Also, we report for the first time the overexpression of three activation markers in ILC2 from humans with ascariasis and the significant correlation between this activation marker expression with egg burden. Finally, using a targeting proteomic approach, we identified different proteins associated with Ascaris infection and one particular mast cell product, TPSAB1, that also correlated with %CD25, %CD69 and % TSLPR+ ILC2s frequencies and, Ascaris IgE levels and egg burden.

To our knowledge, the relationship between *A. lumbricoides* active infection in humans and ILC2 frequencies has not been reported previously. Although Darboe et al. (41) studied the association between specific IgG antibodies against *Ascaris* spp. extract with the frequency of ILC2, this is not an informative measure for active infection, but mostly about exposure that may be almost universal in highly endemic populations. In fact, in our study sample, we observed a high frequency of IgG seropositive response against Ascaris extract or ABA-1 in both, actively infected and non-infected subjects. As we have previously published, in this rural town of Colombia, a prevalence of 63% of ascariasis have been found. Then, it is expected that most individuals are exposed during lifetime to this parasite (7, 29). In our study, ILC2 numbers and frequencies were not different between infected and non-infected subjects; however, we did observe that ILC2s were more abundant in relation to ILC1 and ILC3 in the NI controls but did not in the infected group. Similar to other reports (41), we found ILC2s represented the 50% of ILCs in NI group, however, this significant difference in the distribution disappears in the infected subjects. Comparison of ILC2s in regard to helminth infection have yielded different results. IL-13-producing c-Kit+ ILCs were described as higher in the peripheral blood of adult individuals with filarial infection (20). In contrast, Nausch et al. reported a decrease of the ILC2 population in children infected with *Schistosoma haematobium*, which could be restored and become activated (reflected by the overexpression of TSLP) after anti-helminth treatment (19). Also, Tamadaho et al. found that non-infected subjects had higher number of ILC2 in comparison with patients with active *W. bancrofti* infection (lymphatic filariasis), and this distribution changed after pathogen clearance using deworming treatment (42). Our results are similar to the last two studies. During active infection, it is possible that cells migrate to tissue due to the production of alarmins and chemokines by epithelial cells in the gut, which could result in a reduction of circulating cell numbers. Notably, blood levels of granulocytes and ILC2 mediators (MPO and VEGFA) (43), are strongly and negatively correlated with the relative counts of ILC2 in the blood. This suggests that during infection, ILC2 cells may become activated and migrate to the infected tissues.

Even though, ILC2 tends to decrease in infected individuals, two groups could be identified in this population, one with high ILC2 numbers and the other one with low numbers. IgE sensitization to ABA-1 seems to be associated with the increase of ILC2s in the high group. IgE sensitization against ABA-1 has been reported as a marker of resistance to infection (44) and a factor associated with asthma and its severity (7, 45). In this context, it can be thought that the subjects who have a higher frequency of ILC2 tend to have a stronger T2 response to helminth infection and, similarly, higher ILC2 may contribute to the worsening of allergic diseases (46).

The activation markers CD25, CD69, and TSLPR were significantly more prevalent on ILC2 cells in AI individuals, reinforcing the association between ILC2 activation and active Ascaris infection. Several studies support that upon stimulation with alarmins and cytokines, ILC2 activates and expresses CD25, CD69 or TSLPR. In spite we did not confirm that expression of these surface markers is accompanied by type 2 cytokine production, there are studies that support that these phenotypes are related to enhanced activity in ILC2s. CD25+ ILCs and ILC2s produced higher amounts of type 2 cytokines (47, 48). Also, BAL fluid-derived ILC2s exhibited increased expression of IL-13, AHR, interferon regulatory factor 4 (IRF4), IL-33 receptor ST2 (IL1RL1), and several activation markers, including CD69 (49). Finally, ILC2s from individuals with eosinophilic asthma demonstrated increased TSLPR expression and cytokine release of IL-5 and IL-13 (50).

Moreover, molecules mainly related to granulocyte chemotaxis, tissue remodeling and cell growth were upregulated in AI individuals. Similar to our results, MMP-1, PLXDC1 and GP6 signaling pathways appear to be dysregulated after the stimulation of hepatic stellate cells with two peptides from Sm16, a product from *S. mansoni* (51). Additionally, Ascaris infection also increased circulating levels of JAM-A, a tight junction molecule that regulates the permeability of the epithelium (52), suggesting a possible increase in epithelial permeability due to the loss of this protein from the barrier cells to the circulation. As observed in our findings regarding CXCL6 and CXCL1, chemokines associated with granulocyte migration, like CXCL5, have been reported to be increased in serum from subjects infected with *A. lumbricoides* (53). Granulocytes play a fundamental role in the immune response against pathogens, and in the case of helminths, eosinophils are characterized by leading the fight against and expulsion of these parasites. Interestingly, neutrophil migration pathways are upregulated in subjects with ascariasis. The role of these cells in the defense against this helminth has been scarcely explored in humans. An early intestinal neutrophilic response has been found in a model of mice infected with *Ascaris* spp (54). and there is evidence that neutrophils can destroy larvae of *Ascaris suum* (55). Interestingly, a preliminary study in subjects living in Santa Catalina found lower relative numbers of neutrophils in individuals with ascariasis compared to sex- and age- matched, non-infected control subjects (56), which may be related to an increased migration to tissues where parasites reside (i.e. the gut or the lung).

TPSAB1 is an enzyme associated with mast cell regulation and function. Tryptase plays a role in immune responses and is involved in various physiological and pathological processes, including inflammation, mast cells disorders and allergic reactions (57, 58). Our findings regarding higher TPSAB1 levels indicates that mast cells may be one of the initial and essential sensors during Ascaris infection, which may indirectly activate ILC2s and boost the T2 response. Similar to our results, Ascaris-infected pigs had significantly increased expression of mast cell tryptase, TPSAB1 together with several T2-related molecules (59). These “feed-forward loop” of mast cell activation and T2 response have been supported in airway epithelium in asthma, where the T2 inflammation is associated with the expression of a specific set of mast cell genes (60). Additionally, mast cells seem to be critical in the induction of ILC2 and helminth expulsion as a main source of IL-33 (61). Interaction of mast cells and ILC2 remains to be further studied, as well as the functional role of these cells in infection and resistance to parasites. Our finding of an increase of TPSAB1 has also been reported in eosinophilic chronic obstructive pulmonary disease (62), suggesting that an interaction between ILC2, mast cells and eosinophilic inflammation could be present in ascariasis.

Additional support to this T2 boost is the negative correlation of IL-10 levels with ILC2 frequency. It has been demonstrated that ILC2s can switched to IL-10 regulatory innate lymphoid cells under specific conditions or cytokine milieu (63, 64), so it would be interesting to understand this negative correlation in helminthiasis as compared to allergen immunotherapy where the high dose of allergen could lead to another mechanism of immune regulation.

Several limitations must be acknowledged in this study. The sample size is small, which reduces the power to detect associations. The activation status of ILC2s was not confirmed by measuring type 2 cytokine production, mainly due to limitations in the amount of biological material available. Additionally, it is important to note that under cell culture conditions, cells might develop phenotypes that are induced and do not accurately represent the *in vivo* circulating status of ILC2 cells, which was the main objective of this study. Other surface markers, such as ST2, IL17RB, KLRG1, ICOS, CCR6, and MHCII, as well as transcription factors like GATA3, were not evaluated in the ILC2 subset. Finally, despite its high sensitivity, PEA could not detect type 2 cytokines and alarmins in circulation, which limits our ability to draw conclusions about the systemic type 2 response and its correlation with circulating ILCs.

In summary, this study demonstrates the increase of circulating ILC2 with an activated phenotype and higher TPSAB1 levels associated with *A. lumbricoides* infection, supporting the role of ILC2 in a network of interacting with other cells through increased cytokines and chemokines in the boosting of the T2 immune response and helminth immunity.

## Data Availability

The raw data supporting the conclusions of this article will be made available by the authors, without undue reservation.
