# Supplementary material for: Elevated circulating group-2 innate lymphoid cells expressing activation markers and correlated tryptase AB1 levels in active ascariasis
Source: Front Immunol. 2024 Oct 25;15:1459961. doi: 10.3389/fimmu.2024.1459961 (PMC11549673; doi:10.3389/fimmu.2024.1459961)
Supplement: Supplementary file 1 [file DataSheet1.docx]

Supplementary Material

Elevated circulating group-2 innate lymphoid cells expressing activation markers and correlated tryptase AB1 levels in active ascariasis

Juan-Felipe Lopez^1,2^, Josefina Zakzuk^2^, Pattraporn Satitsuksanoa^1^, Ana Lozano^2^, Laura Buergi^1^, Anja Heider^1^, Juan Carlos Alvarado-Gonzalez^2^, Huseyn Babayev^1^, Cezmi Akdis^1^, Willem van de Veen^1^, Luis Caraballo^2^, Mübeccel Akdis^1*^

^1^Swiss Institute of Allergy and Asthma Research (SIAF), University of Zurich, Davos, Switzerland

^2^Institute for Immunological Research, University of Cartagena, Cartagena, Colombia.

*** Correspondence:**Mübeccel Akdis, MD, PhD

[muebeccel.akdis@siaf.uzh.ch](mailto:muebeccel.akdis@siaf.uzh.ch)

Swiss Institute of Allergy and Asthma Research (SIAF), University of Zurich, Herman-Burchard-Strasse 9, CH-7265 Davos-Wolfgang, Switzerland

# Supplementary Figures Legends

**Supplementary Figure 1. Gating strategy for identification of ILC in PBMCs.** (A)Total viable leukocytes(CD45+) were gated after double discrimination of duplicates. CD127+ ILCs were gated after linage-negative cells were gated. Among the CD127+ gate, positive events for CD161+ were denominated total ILCs. (B) Isotypes and fluorescence minus one (FMO) controls for activation markers**.**

**Supplementary Figure 2. Correlation of %ILC2 and activated phenotypes with serum biomarkers of inflammation and immune response.** In this correlogram, the sets of proteins significantly correlated (*uncorrected p<0.05) with each analyzed ILC2 population (%ILC2, CD25+ ILC2, CD69+ ILC2 and TSLPR+ ILC2) are shown. As part of data treatment, ILC2 related populations were log-transformed for normalization. NPX dataset was first filtered for those variables with high number of values below the limit of detection (>40%); then, we applied a random forest function to identify variable with poor contribution to the variance of ILC2 cell counts (%IncMSE<1.2). Then, a total number of 122 proteins from the Olink panels were analyzed by Pearson correlation also using FDR as a multiple testing correction method.

**Supplementary Figure 3. Protein profile belonging to inflammation and type 1 response, type 2 response, and immune regulation pathways from AI compared to NI individuals.** Comparisons were made using unpaired T-test. ns: non significant.

**Supplementary Figure 4.** (A) Negative correlation between ILC2 frequencies and IL-10 NPX values. Correlation analysis corresponds to Spearman correlation coefficient (r). (B) Variable importance plot using RF model for Ascaris lumbricoides infection. TPSAB1, GP6 and CD69+ ILC2 were the most important variables in determining infection by mean decrease Gini.

# Supplementary Table 1. Flow cytometry antibodies

| BRAND | TARGET | FLOUROCHROME | CAT Nº | | CLONE | ISOTYPE |
| --- | --- | --- | --- | --- | --- | --- |
| eBioscience | CD25 | BUV737 | 367-0259-42 | | BC96 | mIgG1, kappa |
| BD OptiBuild™ | NKp44 | BV421 | 744299 | | p44-8 | mIgG1, kappa |
| Biolegend | CD45 | BV510 | 304036 | | HI30 | mIgG1, kappa |
| BD OptiBuild™ | TSLPR | BV711 | 752982 | | 1F11/TSLPR | mIgG1, kappa |
| Biolegend | CD161 | BV785 | 339930 | | HP-3G10 | mIgG1, kappa |
| Biolegend | CD11c | FITC | 301604 | | 3.9 | mIgG1, kappa |
| Biolegend | CD34 | FITC | 343504 | | 581 | mIgG1, kappa |
| Biolegend | CD94 | FITC | 305504 | | DX22 | mIgG1, kappa |
| Biolegend | CD303 | FITC | 354208 | | 201A | mIgG2a, kappa |
| Biolegend | CD123 | FITC | 306014 | | 6H6 | mIgG1, kappa |
| Biolegend | FceR1a | FITC | 334608 | | AER-37 | mIgG2b, kappa |
| Biolegend | CD14 | FITC | 325604 | | HCD14 | mIgG1, kappa |
| Biolegend | CD16 | FITC | 302006 | | 3G8 | mIgG1, kappa |
| Biolegend | CD1a | FITC | 300104 | | HI149 | mIgG1, kappa |
| Biolegend | CD3 | FITC | 317306 | | OKT3 | mIgG2a, kappa |
| Biolegend | CD4 | FITC | 300506 | | RTA-T4 | mIgG1, kappa |
| Biolegend | CD8a | FITC | 301006 | | RPA-T8 | mIgG1, kappa |
| Biolegend | CD19 | FITC | 392508 | | 4G7 | mIgG1, kappa |
| Biolegend | CD69 | PE | 310906 | | FN50 | mIgG1, kappa |
| Biolegend | CD117 (c-kit) | PE-Dzl594 | 313226 | | 104D2 | mIgG1, kappa |
| Biolegend | CD127 | PE-Cy7 | 351320 | | A019D5 | mIgG1, kappa |
| Biolegend | CRTH2 | APC | 350110 | | BM16 | rIgG2a, κ |
| Biolegend | Zombie Green | | | 423112 | NA | NA |

# NA: Not applicable; mIgG: Mouse immunoglobin G; rIgG; Rat immunoglobin G
