# Supplementary figures and images for: Elevated circulating group-2 innate lymphoid cells expressing activation markers and correlated tryptase AB1 levels in active ascariasis
Source: Front Immunol. 2024 Oct 25;15:1459961. doi: 10.3389/fimmu.2024.1459961 (PMC11549673; doi:10.3389/fimmu.2024.1459961)

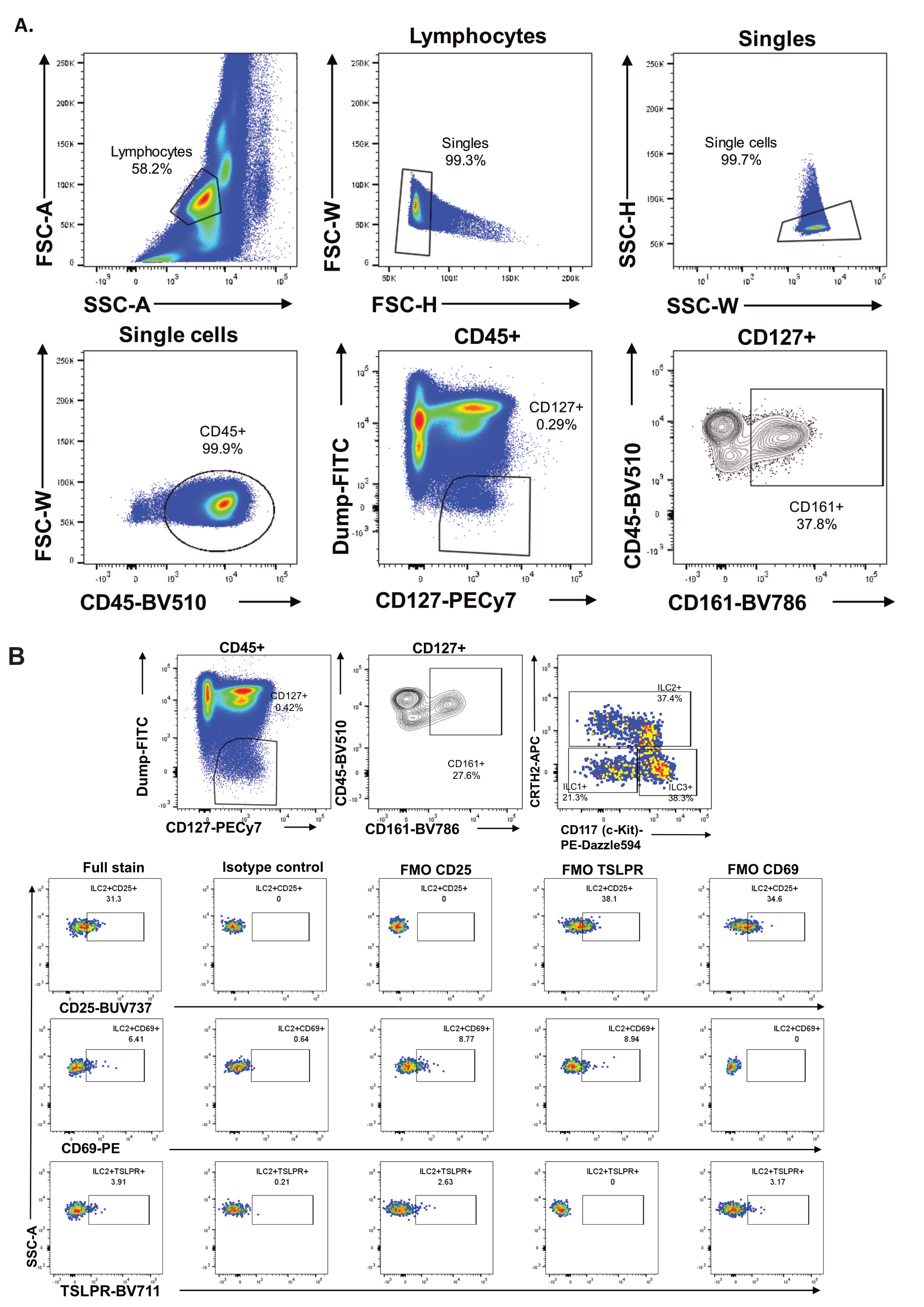

Supplement: Supplementary file 2 [file Image1.tiff]

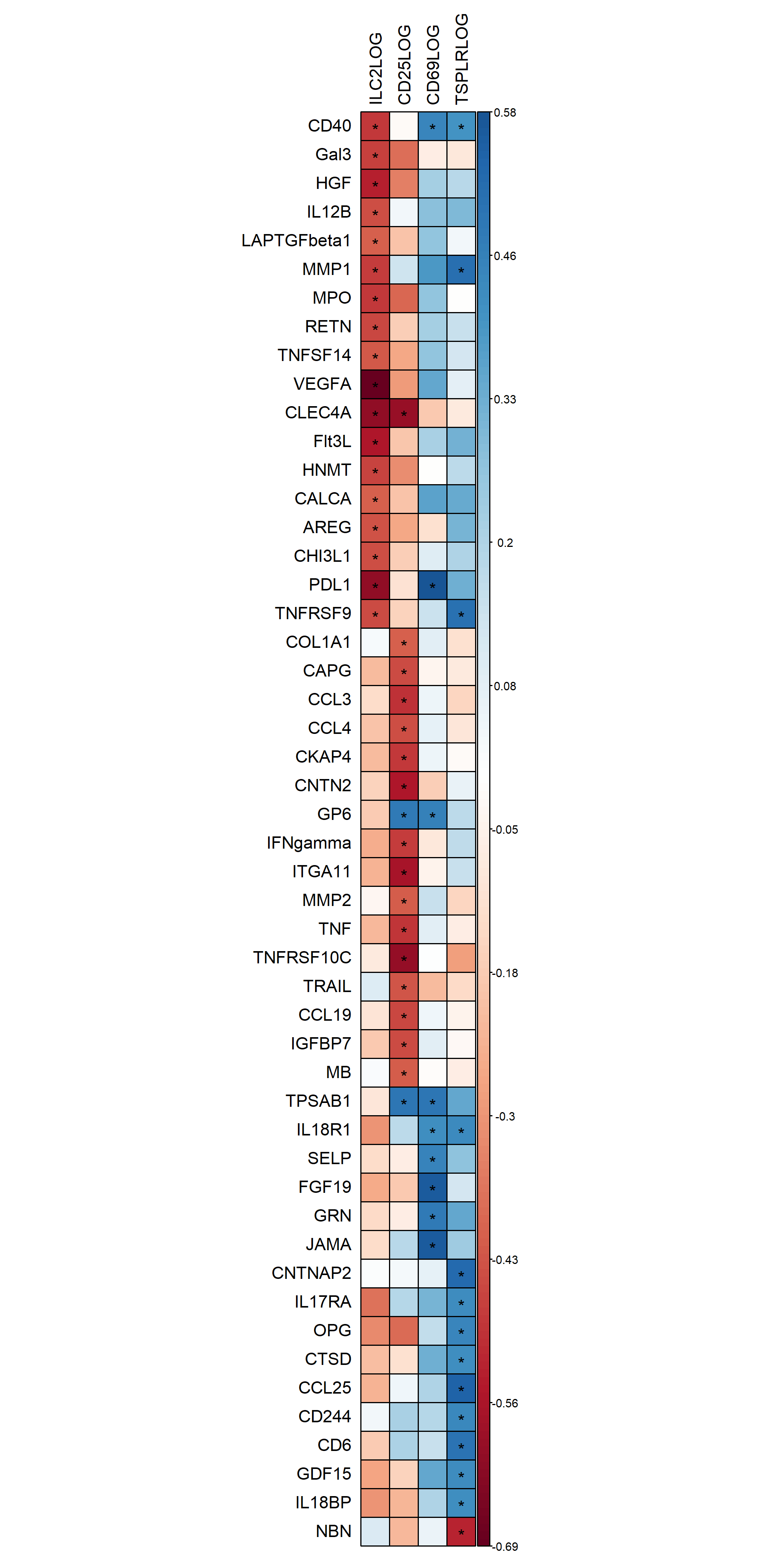

Supplement: Supplementary file 3 [file Image2.tif]

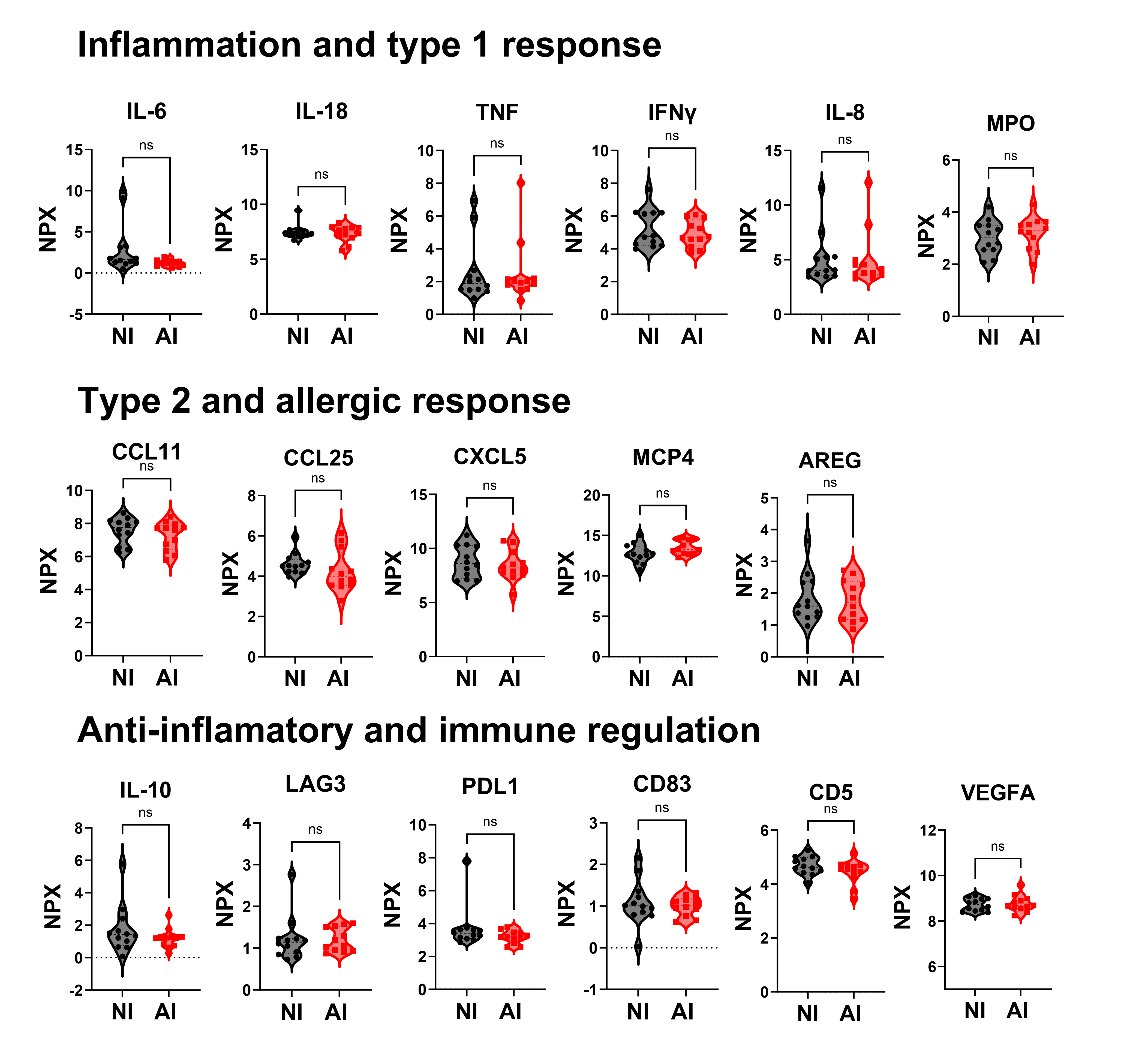

Supplement: Supplementary file 4 [file Image3.tif]

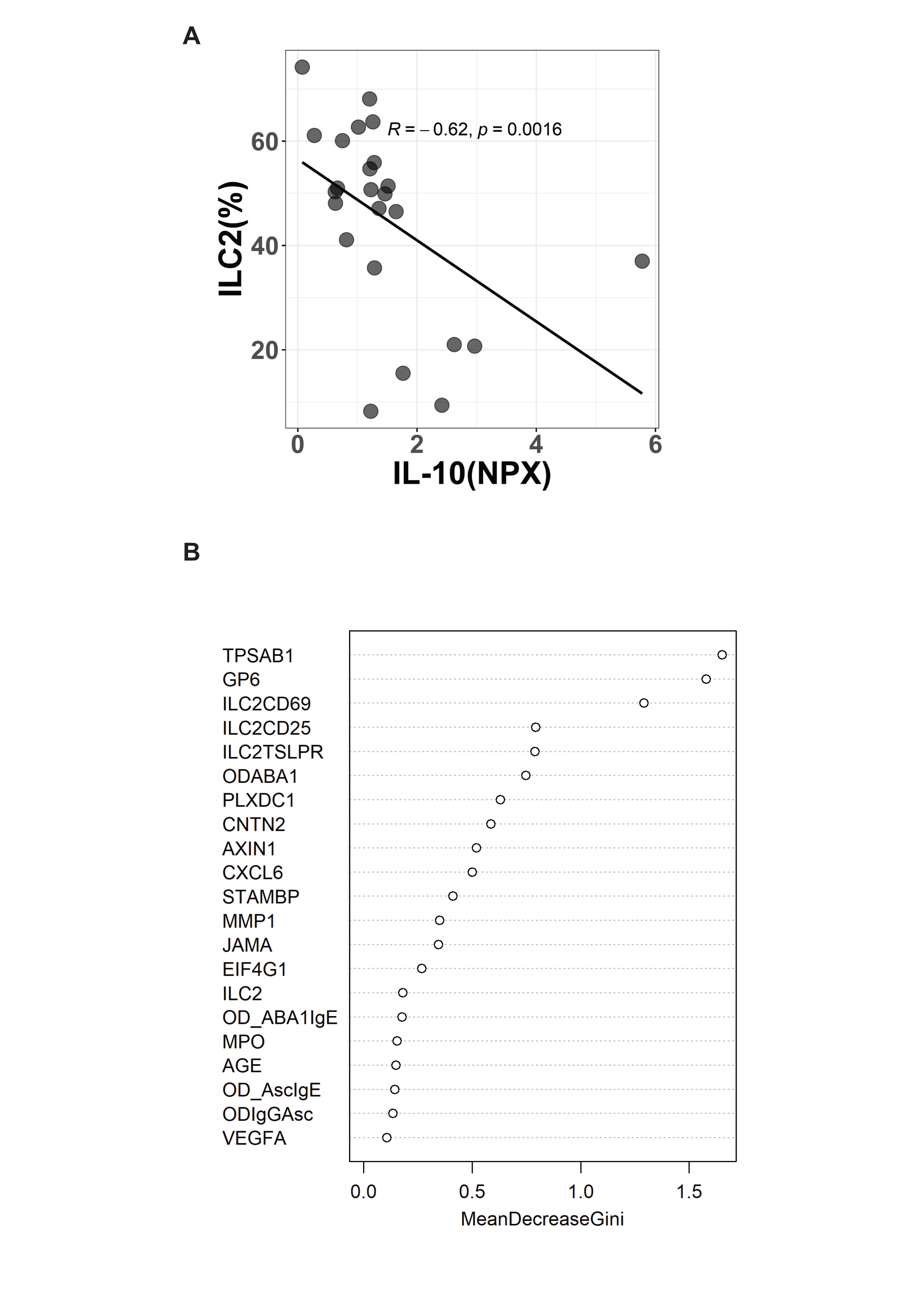

Supplement: Supplementary file 5 [file Image4.tiff]
